# Diagnostic models and predictive drugs associated with cuproptosis hub genes in Alzheimer's disease

**DOI:** 10.3389/fneur.2022.1064639

**Published:** 2023-01-26

**Authors:** Erdong Zhang, Fengqiu Dai, Tingting Chen, Shanhui Liu, Chaolun Xiao, Xiangchun Shen

**Affiliations:** ^1^State Key Laboratory of Functions and Applications of Medicinal Plants, Guizhou Medical University, Guiyang, Guizhou, China; ^2^Key Laboratory of Optimal Utilization of Natural Medicinal Resources, Guizhou Medical University, Guiyang, Guizhou, China; ^3^Department of Anatomy, School of Basic Medical Sciences, Guizhou Medical University, Guiyang, China; ^4^Guiyang Maternal and Child Health-Care Hospital, Guiyang, Guizhou, China; ^5^Key Laboratory of Urological Diseases in Gansu Province, Gansu Nephro-Urological Clinical Center, Institute of Urology, Lanzhou University Second Hospital, Lanzhou, Gansu, China

**Keywords:** cuproptosis, Alzheimer's disease, diagnostic, drug, immune

## Abstract

Alzheimer's disease (AD) is a chronic neurodegenerative disease, and its underlying genes and treatments are unclear. Abnormalities in copper metabolism can prevent the clearance of β-amyloid peptides and promote the progression of AD pathogenesis. Therefore, the present study used a bioinformatics approach to perform an integrated analysis of the hub gene based on cuproptosis that can influence the diagnosis and treatment of AD. The gene expression profiles were obtained from the Gene Expression Omnibus database, including non-demented (ND) and AD samples. A total of 2,977 cuproptosis genes were retrieved from published articles. The seven hub genes associated with cuproptosis and AD were obtained from the differentially expressed genes and WGCNA in brain tissue from GSE33000. The GO analysis demonstrated that these genes were involved in phosphoribosyl pyrophosphate, lipid, and glucose metabolism. By stepwise regression and logistic regression analysis, we screened four of the seven cuproptosis genes to construct a diagnostic model for AD, which was validated by GES15222, GS48350, and GSE5281. In addition, immune cell infiltration of samples was investigated for correlation with these hub genes. We identified six drugs targeting these seven cuproptosis genes in DrugBank. Hence, these cuproptosis gene signatures may be an important prognostic indicator for AD and may offer new insights into treatment options.

## Introduction

Alzheimer's disease is a neurodegenerative disease characterized by aging and irreversibility. It is predicted that dementia prevalence will double in Europe and triple globally by 2050 (1). The number of Americans with Alzheimer's dementia is estimated at 6.5 million over the age of 65 (2). The number of people with AD is forecast to grow to 13.8 million by 2060 without medical breakthroughs to prevent, slow, or treat it (2). According to the Alzheimer's Disease Foundation, Alzheimer's disease is the fifth leading cause of death among Americans aged 65 and older (3). It should be noted that about 5% of patients with AD inherit the disease in a chromosomal-dominant manner, and carriers can develop the disease as late as 40–60 years of age (3). Heritable familial AD (FAD) is most commonly caused by presenilin and the amyloid precursor protein (4). A major pathological feature of AD is the deposit of extracellular amyloid plaques and neurofibrillary tangles (NTFs) in the brain (5). According to the consensus, tau and amyloid β-protein (Aβ) are both essential to the neurodegenerative process in AD, and Aβ is located upstream of tau in the pathway (5). There is evidence indicating that amyloid plaque is not as detrimental as the soluble oligomeric form of Aβ, which causes synaptic dysfunction (6). However, the exact pathogenic form of Aβ and the molecular mechanism by which pathogenic Aβ causes subsequent synaptic and neurotoxic processes are yet unclear (7). Currently, the U.S. Food and Drug Administration (FDA) has licensed six medications (including donepezil, rivastigmine, galantamine, minocycline, and donepezil) to temporarily treat Alzheimer's symptoms without affecting the underlying brain changes or changing the course of the illness (3). Consequently, finding novel, potent targeted agents is urgently needed to further AD diagnosis and care.

In addition to Aβ and NTFs, neuroinflammation plays a significant role in the development of AD (1). It has been shown that Aβ causes microglia to release cytokines that pass through the blood-brain barrier with increasing age, leading to an accumulation of Aβ (8–10). Aβ-activated microglia secrete neurotoxic cytokines and chemokines (TNF, IL-6, IL-1, and CCL2), which cause neurological dysfunction and death (11). Clinical studies suggest anti-inflammatory cytokines (IL-2, IL-4, and IL-33) can modulate microglia activation and mitigate AD pathology (12, 13). The permeability of immune cells and molecules that pass through the blood-brain barrier increases with age, contributing to neurodegeneration in AD (14). Although a relationship between AD and neuroinflammation has been identified, it is unclear whether it is a cause or a consequence of the disease. Therefore, further research into the relationship between AD and inflammation could enhance our understanding of AD and contribute to developing more effective AD treatments.

A metal with redox activity, copper is involved in several metabolic processes in the brain (15). It serves as the active site for several copper enzymes, including cytochrome oxidase, ceruloplasmin, SOD1, and lysinase (16). The uptake and secretion of intracellular and extracellular copper ions are mediated by CTR1 and ATP7A/B (17). There is evidence that ATP7B (K832R) mutations may increase the risk of AD (18). A large number of copper ions accumulate in neurons in the brains of patients with Alzheimer's disease, primarily in amyloid plaques and tangles (19). It has been shown that copper ions can reduce copper ions in neurons and tissues of the brain when APP expression is increased (20, 21). The deficiency of intracellular copper ions can promote the production of Aβ, while the accumulation of extracellular copper ions can promote the aggregation of Aβ (22, 23). Copper ions can affect the division and proliferation of neutrophils in the peripheral circulation and have a positive correlation with the severity of AD (24). The neutrophil can cross the blood-brain barrier and aggregate near Aβ plaques in the AD mouse model (25). Depletion of neutrophils enhanced cognitive performance, reduced microglia, and reduced Aβ 1–42 levels in 3xTg-AD mouse brain homogenates (26). Thus, regulation of intracellular and extracellular copper iron transport is still under research for the treatment of AD. As one of the causes of neuronal ROS, copper ions combined with Tau protein can lead to the production of H_2_O_2_ *in vitro*. It was shown that intracellular delivery of copper ions reduced intracellular Tau phosphorylation in a mouse animal model of AD (APP/PS1) (27). Therefore, the relationship between copper ion-related metabolism, immune cells, and molecules and AD is not negligible.

**Figure 1:**
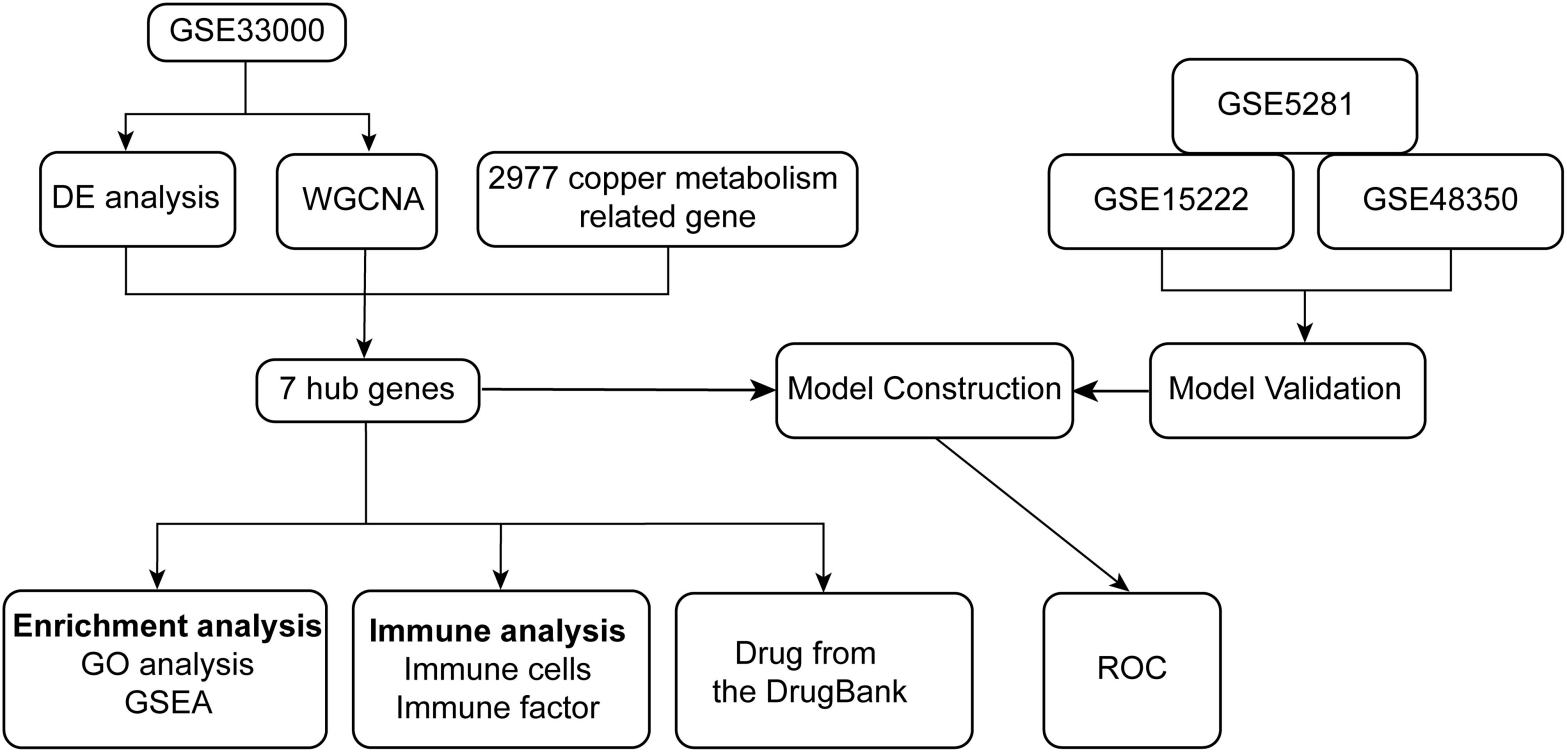
The flowchart of the analyses.

Through the analysis of differentially expressed genes (DEGs) and Weighted gene co-expression network analysis (WGCNA) between ND and AD on the GSE33000 dataset, we identified seven hub genes related to cuproptosis and AD. The biological processes and pathways of seven hub genes have been analyzed using GO and gene set enrichment analysis (GSEA) in patients with AD. We developed a diagnostic model for AD based on stepwise regression and logistic regression analyses and validated it in the GSE15222, GSE48350, and GSE5281 datasets, respectively. It is evident from the AUC value of ROC that the model has good diagnostic performance and may be useful in the diagnosis of AD. Symptoms of neuroinflammation can be accompanied by tangles of tau, which can result from interactions with amyloid plaques. Thus, the significance of immune infiltrating cells for these hub genes was explored in AD samples. Finally, we retrieved drugs targeting seven hub genes from the DrugBank database, which have implications for the treatment of AD. The flowchart of the research is shown in Figure 1.

## Materials and methods

### Data acquisition

In total, 2,977 genes associated with cuproptosis were found in the published literature (28). In this study, all expression profiles were obtained from public databases. Gene expression data were obtained from the GEO database (https://www.ncbi.nlm.nih.gov/geo/). GSE33000 expression data were obtained using the GPL10558 platform, containing 155 ND samples and 310 AD samples. GSE15222 expression data was used with the GPL2700 platform, containing 40 ND samples and 31 AD samples. GSE48350 expression data was used with the GPL570 platform, containing 27 ND samples and 21 AD samples. GSE5281 expression data was used with the GPL570 platform, containing 11 ND samples and 22 AD samples.

### Differential expression analysis

Here, we used the “limma” package (version 3.40.6) for differential analysis to obtain differential genes between the AD and ND groups (29). Specifically, we obtained the expression profile dataset, performed multiple linear regression using the lmFit function, and further used the eBays function to compute moderated *t*-statistics, moderated *F*-statistics, and log-odds of differential expression by empirical Bayes moderation of the standard errors toward a common value, finally obtaining the differential significance of each gene. The differentially expressed genes (DEGs) between AD and AD were filtered with the threshold |logFC| > 1 and adj.*p*.val < 0.05.

### Weighted gene co-expression network analysis

Weighted gene co-expression network analysis is a method for analyzing gene expression patterns of multiple samples (30). It can cluster genes by similar gene expression patterns, form modules, and analyze the relationships between modules and the clinical information of patients (30). First, we calculated the MAD (median absolute deviation) of each gene separately using the gene expression profile, and then we eliminated the top 50% of genes with the smallest MAD, removed the outlier genes and samples using the goodSamplesGenes method of the R package WGCNA, and further constructed a scale-free co-expression network using WGCNA. First, Pearson's correlation matrices and the average linkage method were both performed for all pair-wise genes. Then, a weighted adjacency matrix was constructed using a power function A_mn_ = |C_mn_|^β^ (C_mn_ = Pearson's correlation between Gene_m_ and Gene_n_; A_mn_ = adjacency between Gene_m_ and Gene_n_). β was a soft-thresholding parameter that could emphasize strong correlations between genes and penalize weak correlations. After choosing the power of 7, the adjacency was transformed into a topological overlap matrix (TOM), which could measure the network connectivity of a gene defined as the sum of its adjacency with all other genes for network generation, and the corresponding dissimilarity (1-TOM) was calculated. To classify genes with similar expression profiles into gene modules, average linkage hierarchical clustering was conducted according to the TOM-based dissimilarity measure with a minimum size (gene group) of 30 for the gene dendrogram. The sensitivity is 3. To further analyze the module, we calculated the dissimilarity of module eigengenes, chose a cut line for the module dendrogram, and merged some modules. In addition, we merged modules with distances < 0.25 and finally obtained 13 co-expression modules. Notably, the gray module was considered a set of genes that could not be assigned to any module. Finally, we calculated the correlation between module vectors and gene expression to obtain MM (MM >0.8), and 206 genes with high connectivity in the clinically significant module were identified as hub genes.

### Identification of hub genes

To obtain genes related to cuproptosis genes and AD, DEGs, intersections of genes obtained by WGCNA and cuproptosis genes were taken using the “VeenDiagram” package in R software. The differential expression of the hub gene in ND and AD was represented using violin plots. The hypothesis tests used were the *t*-test and the Mann–Whitney *U*-test. The former was used if the data conformed to a normal distribution, and the latter if not. Significance was defined as *p* < 0.05.

### Enrichment analysis

To investigate the biological mechanisms affecting the hub gene for AD, we performed a functional enrichment analysis. We first analyzed the biological process (BP) of gene ontology (GO) in which these genes are involved and presented the final results as a chord plot using the “GOplot” package in the R software. We obtained the GSEA software (version 4.3) from the GSEA (http://software.broadinstitute.org/gsea/index.jsp) website, divided the samples into high (≥50%) and low (< 50%) expression groups based on the expression levels of hub genes, and downloaded the c2.cp.kegg.v7.5.1.symbols.gmt subset from the Molecular Signatures Database (http://www.gsea-msigdb.org/gsea/downloads.jsp); the c2.cp.kegg.v7.5.1.symbols.gmt subset was downloaded to evaluate the relevant pathways and molecular mechanisms based on gene expression profiles and phenotypic groupings, setting a minimum gene set of 5 and a maximum gene set of 5,000, with 1,000 resamples. Screening conditions of *p* < 0.05 and FDR < 0.25 were considered to be statistically significant.

We used a query of multiple protein names (“IFI30,” “CLIC1,” “LYZ,” “PYGL,” “PLA1A,” “ALOX5AP,” and “A4GALT”) and organisms (“*Homo sapiens*”) to search the STRING website (https://string-db.org/). Following that, we set the following main parameters, namely, network type (“full STRING network”), network edge meaning (“evidence”), active interaction sources (“experiments”), the minimum required interaction score [“low confidence (0.150)”], and the maximum number of interactors to show (“no more than 50 interactors” in the first shell). Finally, the available experimentally determined binding proteins were obtained and visualized by Cytoscape software (version 3.9.1).

### Signature for patients with AD

A logistic model is a statistical model that simulates the probability of an event by making the logarithm of the event a linear combination of one or more independent variables and is often used in disease diagnosis (31). In this study, logistic regression with two response variables was used, with 1 representing the AD sample and 0 representing the ND sample. Stepwise regression analysis was used to eliminate factors that were not significant for the response variable and only those that were significant were retained to simplify the model. Stepwise regression iteratively adds or removes variables from the model until the statistical value of the Akaike information criterion (AIC) is minimized. After that, logistic regression was used to fit the relationship between these significant factors and the response variables.

Finally, we performed ROC analysis using the R package pROC (version 1.18.0) to obtain the AUC. Specifically, we obtained the patient's risk score, performed ROC analysis using the roc function of pROC, and evaluated the AUC and confidence interval using the ci function of pROC to obtain the final AUC result of 0.91.

### Immune infiltration

CIBERSORT, a deconvolution algorithm, was used to estimate 22 types of infiltration immune cells in patients with ND and AD by using the normalized gene expression (32). In this study, we analyzed the abundance of infiltrating immune cells in ND and AD tissues using CIBERSORT of the LM22 gene file, including plasma cells, naïve B cells, memory B cells, memory CD4^+^ T cells (activated and resting), naïve CD4^+^ T cells, CD8^+^ T cells, follicular helper T cells, γδ T cells, Tregs, macrophages (M0, M1, and M2), mast cells (activated and resting), dendritic cells (activated and resting), NK cells (activated and resting), neutrophils, monocytes, and eosinophils.

### Drugs from the DrugBank

DrugBank (www.drugbank.ca) is a comprehensive, freely available web resource containing detailed drug, drug target, drug action, and drug interaction information on FDA-approved drugs and experimental drugs undergoing the FDA approval process (33). In this research, the “Targets” module of the DrugBank database was used to analyze the seven hub genes of drug targeting.

### Statistical analysis

All statistical analyses in this study were performed using the R language. The *t*-test and the Mann–Whitney *U*-test were selected according to whether the data conformed to a normal distribution. Statistical significance was defined as *p* < 0.05.

## Results

### Identification of hub genes for AD and cuproptosis

**Figure 2:**
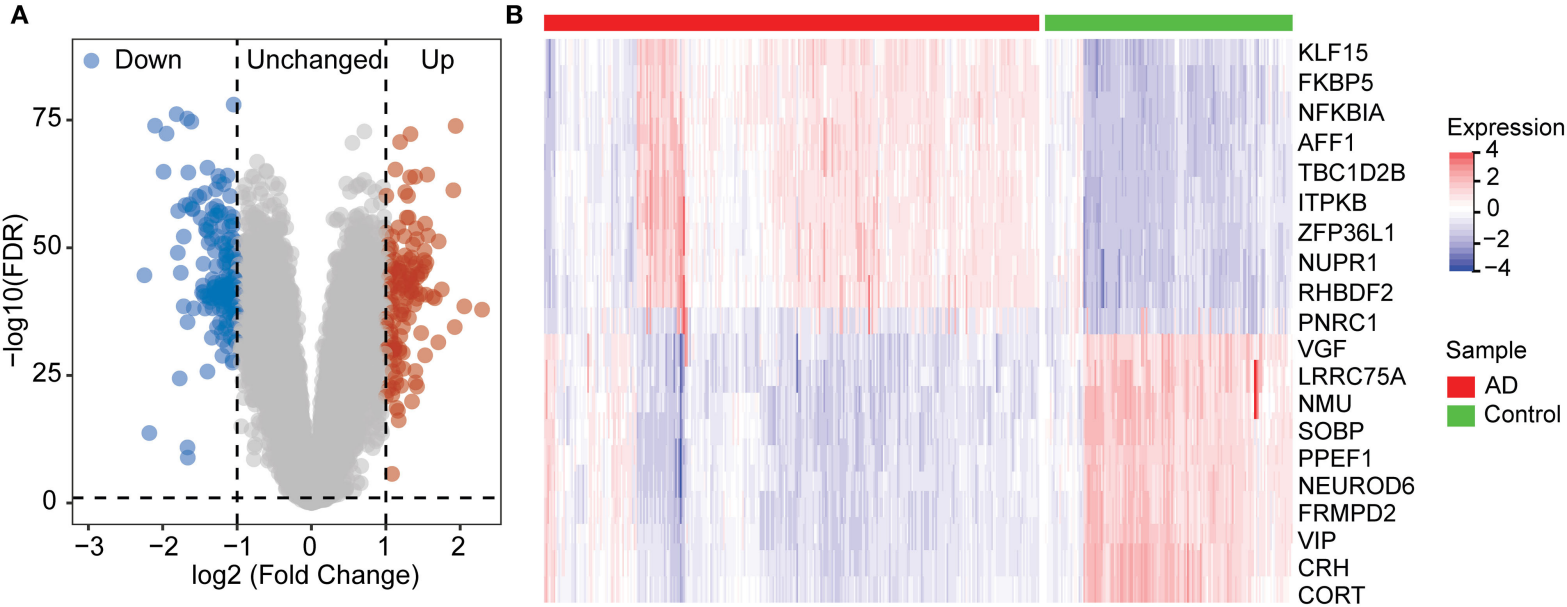
Differentially expression gene between ND and AD samples. **(A)** Volcano plot showing the significant genes found by limma analysis. Red genes represent significantly high expression in AD, blue genes indicate significantly high expression in ND, and gray genes mean no changes. **(B)** The top 20 genes significantly expressed in AD or ND samples, showing by the heatmap.

To identify genes associated with AD, we analyzed a total of 299 differential genes between ND and AD in the GSE33000 dataset using the limma package with the screening criteria of adj.*p*.val < 0.05 and |logFC|> 1, including 139 upregulated genes and 160 downregulated genes (Supplementary Table S1). Expression differential genes were presented in a volcano plot (Figure 2A), and heat maps of the top 10 differentially expressed genes that were upregulated and downregulated, respectively, were plotted (Figure 2B).

**Figure 3:**
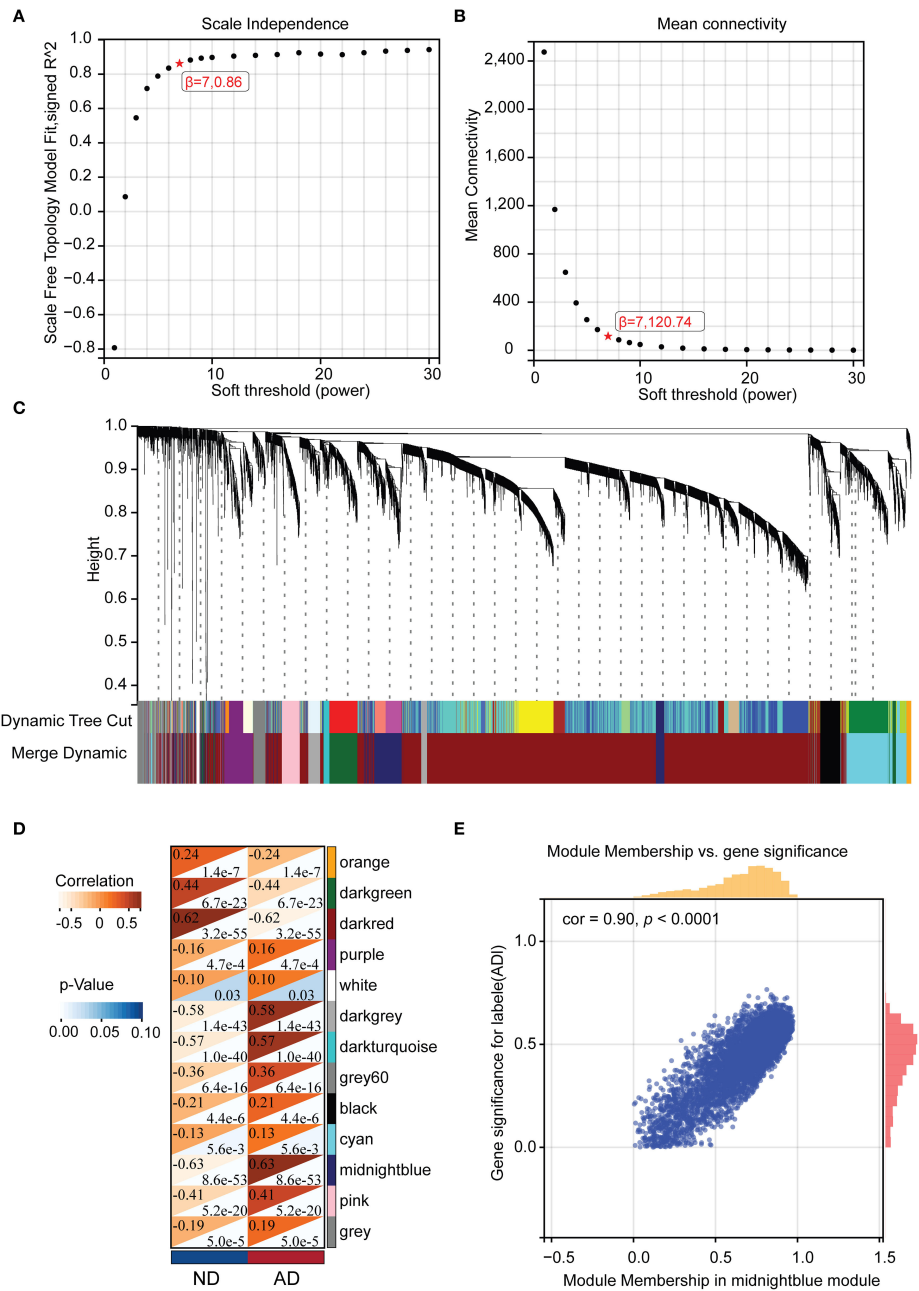
The WGCNA results. **(A)** Analysis of the scale-free fit index for various soft-thresholding powers (β). **(B)** The mean connectivity for the soft-thresholding powers. **(C)** Clustering dendrograms of genes, with dissimilarity based on the topological overlap, together with assigned module colors. **(D)** Correlations between different modules and clinical traits. **(E)** Correlation of module membership and gene significance in the midnight blue module.

After removing outlier genes and samples using the goodSamplesGenes method in the WGCNA package, the expression profiles of 7,595 genes and 465 samples were taken from GSE33000 for constructing a weighted gene co-expression network (soft threshold force: 7, scale independence: 0.86, average linkage: 120.74, Figures 3A, B). The 13 different co-expression modules were obtained through dynamic tree cutting (module merging threshold: 0.25, minimum module: 30, Figure 3C). The correlation analysis was then performed for each module with clinical traits. It was shown that the midnight blue (*r* = 0.64, *p* = 8.6e-53) and dark red module (*r* = −0.62, *p* = 3.2e-55) had the highest positive and negative correlation with AD, respectively (Figure 3D). Thus, we selected the midnight blue module (containing 453 genes) with the highest correlation coefficients for further analysis. GS- and MM-related scatterplots showed that these genes were highly correlated with both modules and phenotypes (cor = 0.90, *p* < 0.001; Figure 3E).

**Figure 4:**
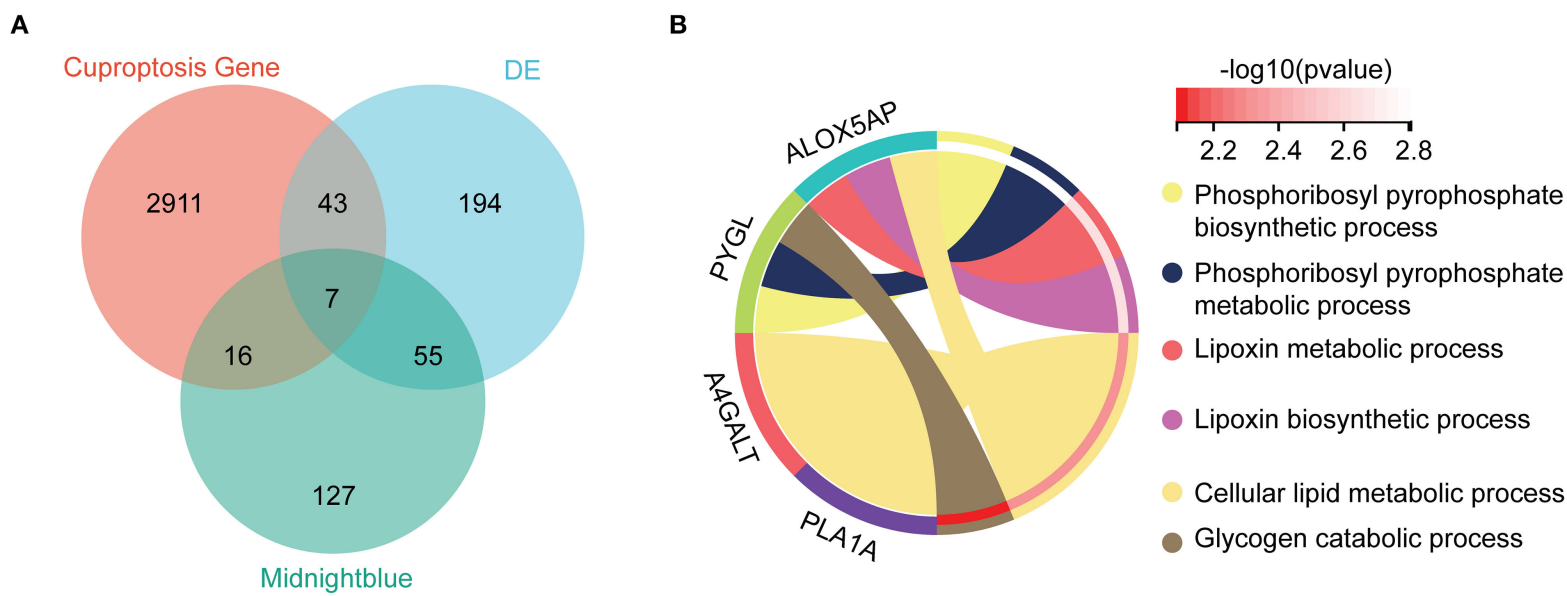
Hub genes and GO analysis. **(A)** The intersections of the DE, cuproptosis genes, and midnight blue genes. **(B)** Biological processes in which the seven hub genes were involved.

**Figure 5:**
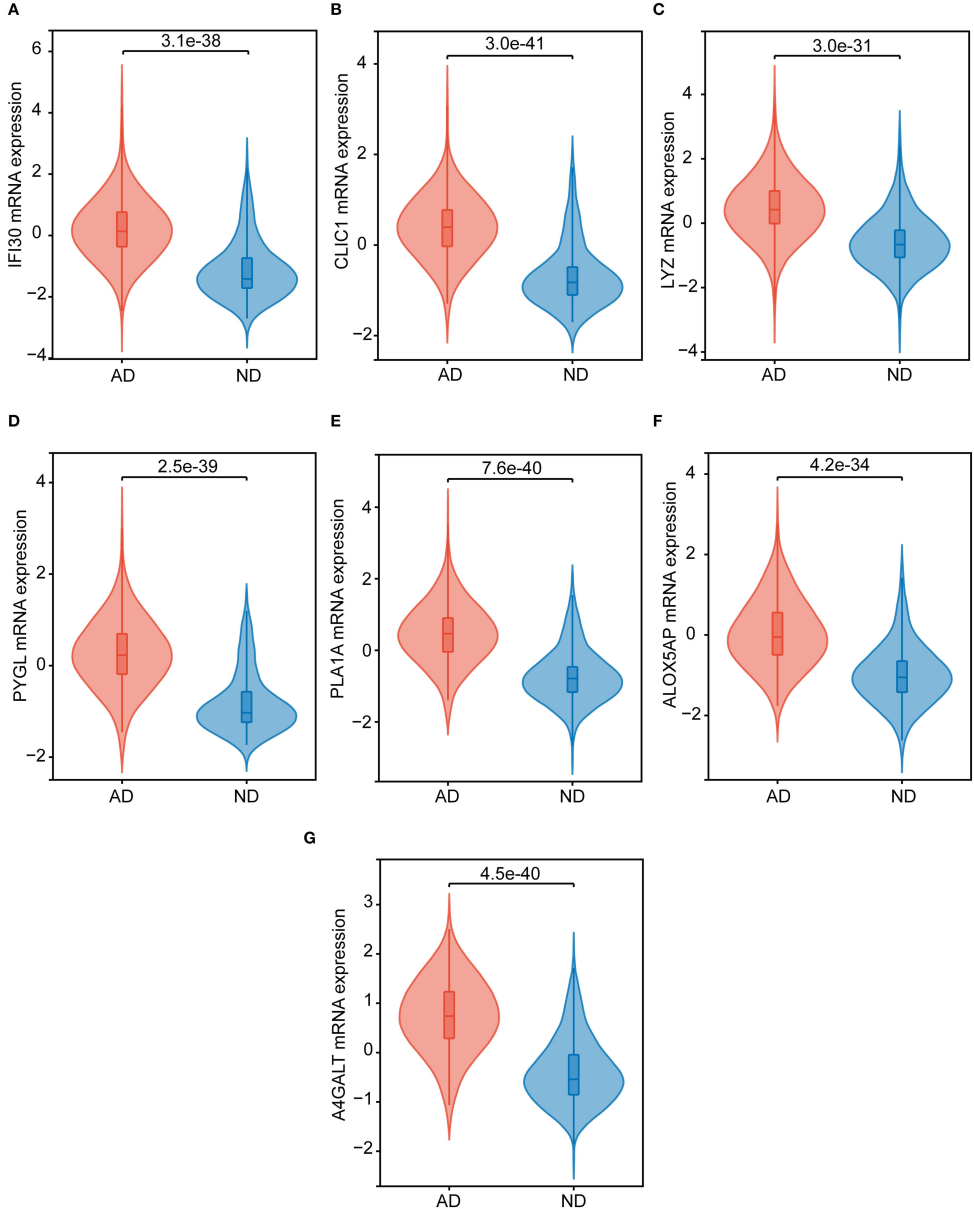
The hub genes expression in the ND and AD samples of GSE33000. **(A)** IFI30, **(B)** CLIC1, **(C)** LYZ, **(D)** PYGL, **(E)** PLA1A, **(F)** ALOX5AP, and **(G)** A4GALT.

By taking the intersection of 299 differential genes in GSE33000, 453 genes in the midnight blue module of WGCNA, and 2,977 genes related to cuproptosis genes, we finally obtained the seven hub genes of cuproptosis associated with AD (Figure 4A). The results of the violin plot showed that the seven hub genes were highly expressed in patients with AD (*p* < 0.05, Figure 5). In addition, the expression of these genes in GSE15222, GSE48350, and GSE5281 is shown in Supplementary Figure S1.

### Enrichment for the hub gene

To further investigate the molecular mechanism of the seven hub genes in AD, we attempted to screen out the targeting of the hub gene binding protein. Based on the STRING tool, we found a total of binding proteins, which were validated by experimental data (Supplementary Figure S2). We performed an enrichment analysis to investigate the potential biological role of these hub genes. The GO analysis showed that four of the seven hub genes were involved in biological processes (BP), including phosphoribosyl pyrophosphate biosynthetic process, phosphoribosyl pyrophosphate metabolic process, lipoxin metabolic process, lipoxin biosynthetic process, cellular lipid metabolic process, and glycogen catabolic process (Figure 4B).

**Figure 6:**
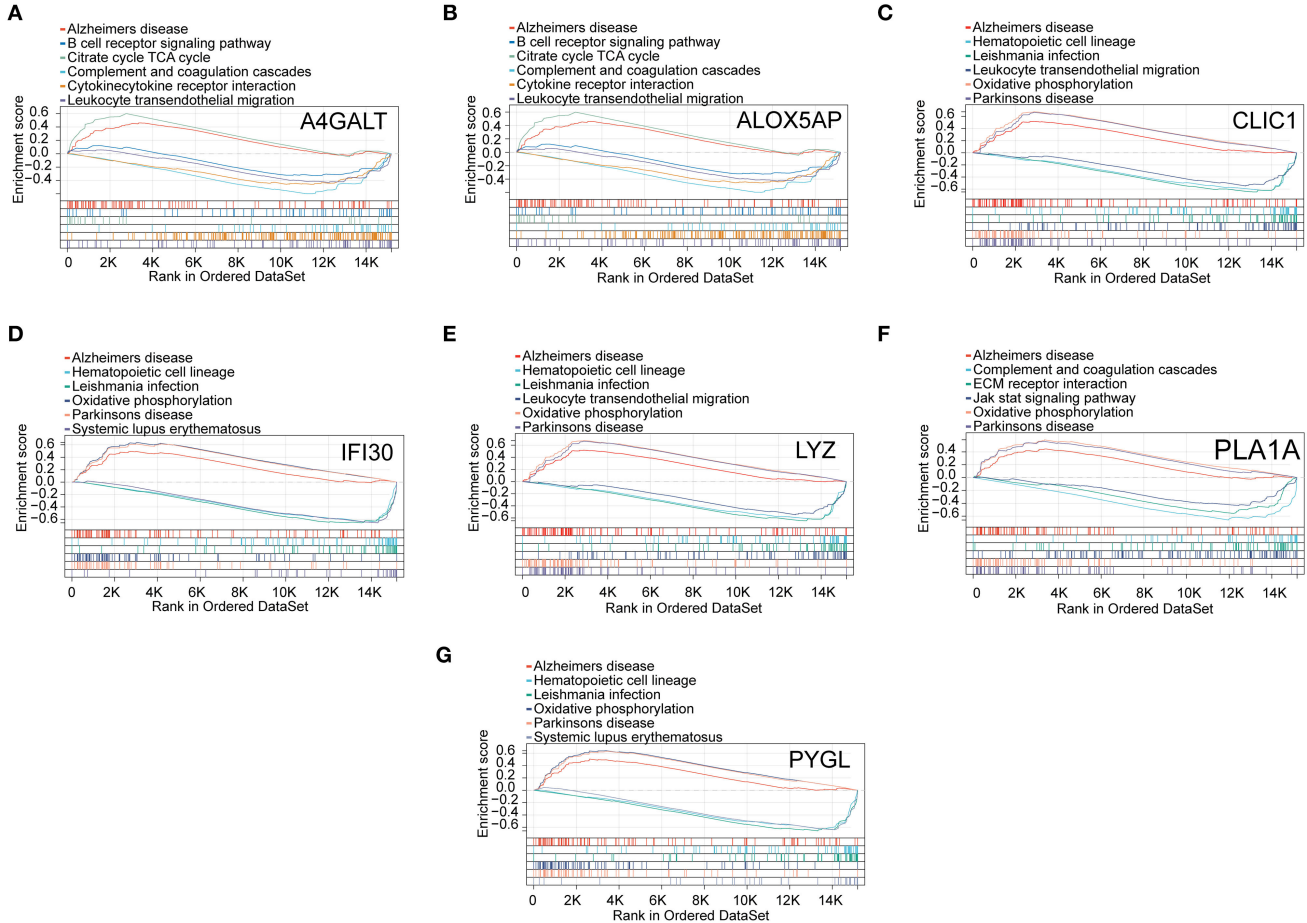
GSEA revealed the enriched pathways of the hub genes. **(A)** A4GALT, **(B)** ALOX5AP, **(C)** CLIC1, **(D)** IFI30, **(E)** LYZ, **(F)** PLA1A, and **(G)** PYGL.

The results of the GSEA analysis showed that these hub genes were associated with neurodegenerative diseases (AD and Parkinson's disease), oxidative phosphorylation, leukocyte transendothelial migration, leishmania infection, hematopoietic cell lineage, complement and coagulation cascades, citrate cycle (TCA cycle), B cell receptor signaling pathway, systemic lupus erythematosus, cytokine receptor interaction, ECM receptor interaction, and Jak stat signaling pathway (Figure 6).

### Construction and validation of diagnostic models

**Figure 7:**
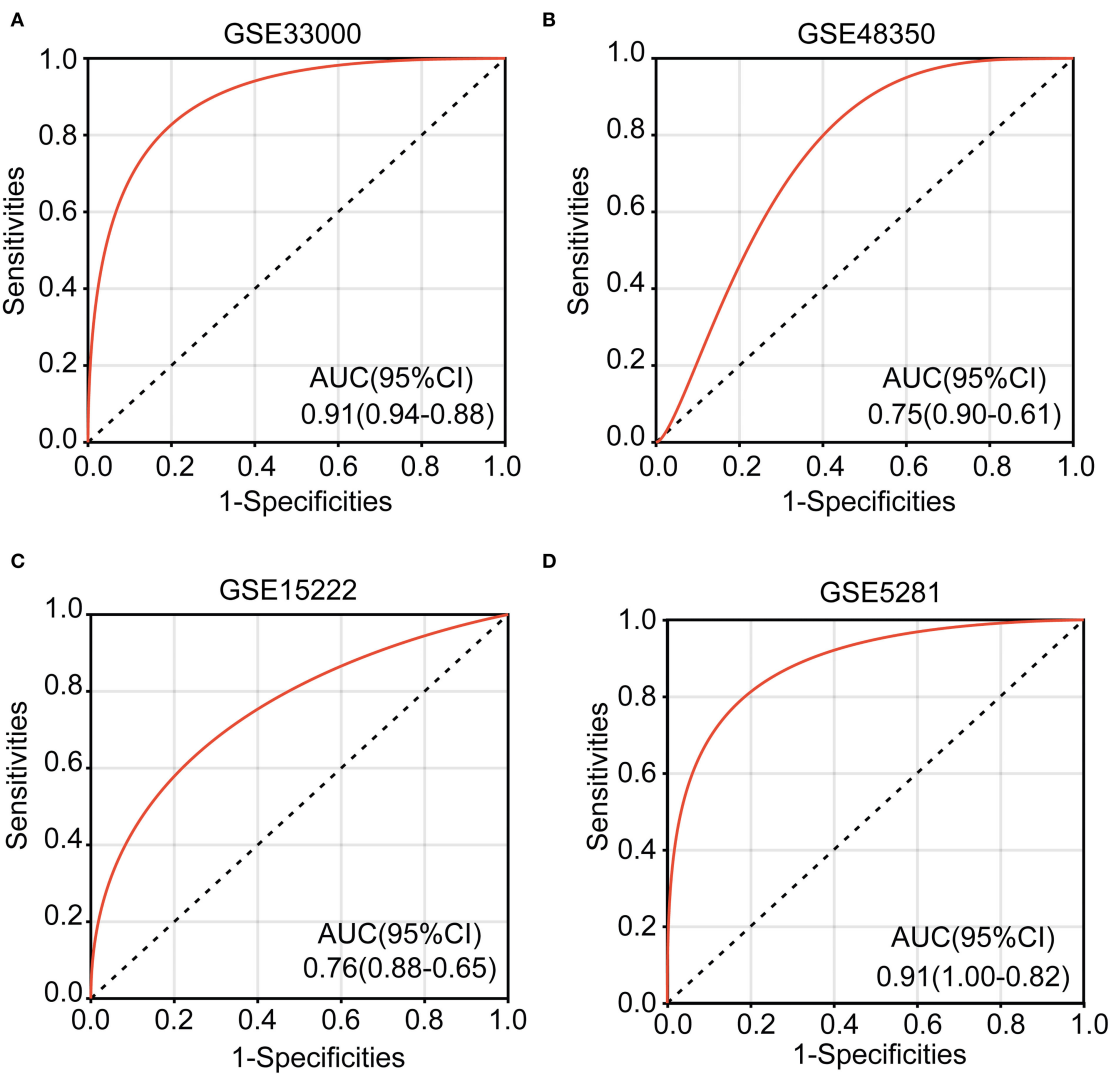
Receiver operating characteristic (ROC) curves and corresponding AUC values for the four expression cohorts. **(A)** GSE33000, **(B)** GSE48350, **(C)** GSE15222, and **(D)** GSE5281.

Logistic regression was used to perform a multi-gene prediction model based on GSE33000. Logistic regression was used to perform a multi-gene prediction model based on GSE33000. The prediction model was constructed for four of the seven cuproptosis genes, including *IFI30, PLA1A, ALOX5AP*, and *A4GALT*. The results showed that the constructed model had good diagnostic performance, and the area under the ROC curve was 0.91 (Figure 7A). We used the GSE48350, GSE15222, and GSE5281 datasets for validation, and the results showed that the area under the ROC curve was 0.75, 0.76, and 0.91, respectively (Figures 7B–D). The formula was as follows: [expression level of *IFI30* × (−0.7449)] + [expression level of *PLA1A* × 0.6557] + [Expression level of *ALOX5AP* × 1.1483)] + [expression level of *A4GALT* × 1.4343] + 0.8891.

### Immune infiltration in patients with AD

**Figure 8:**
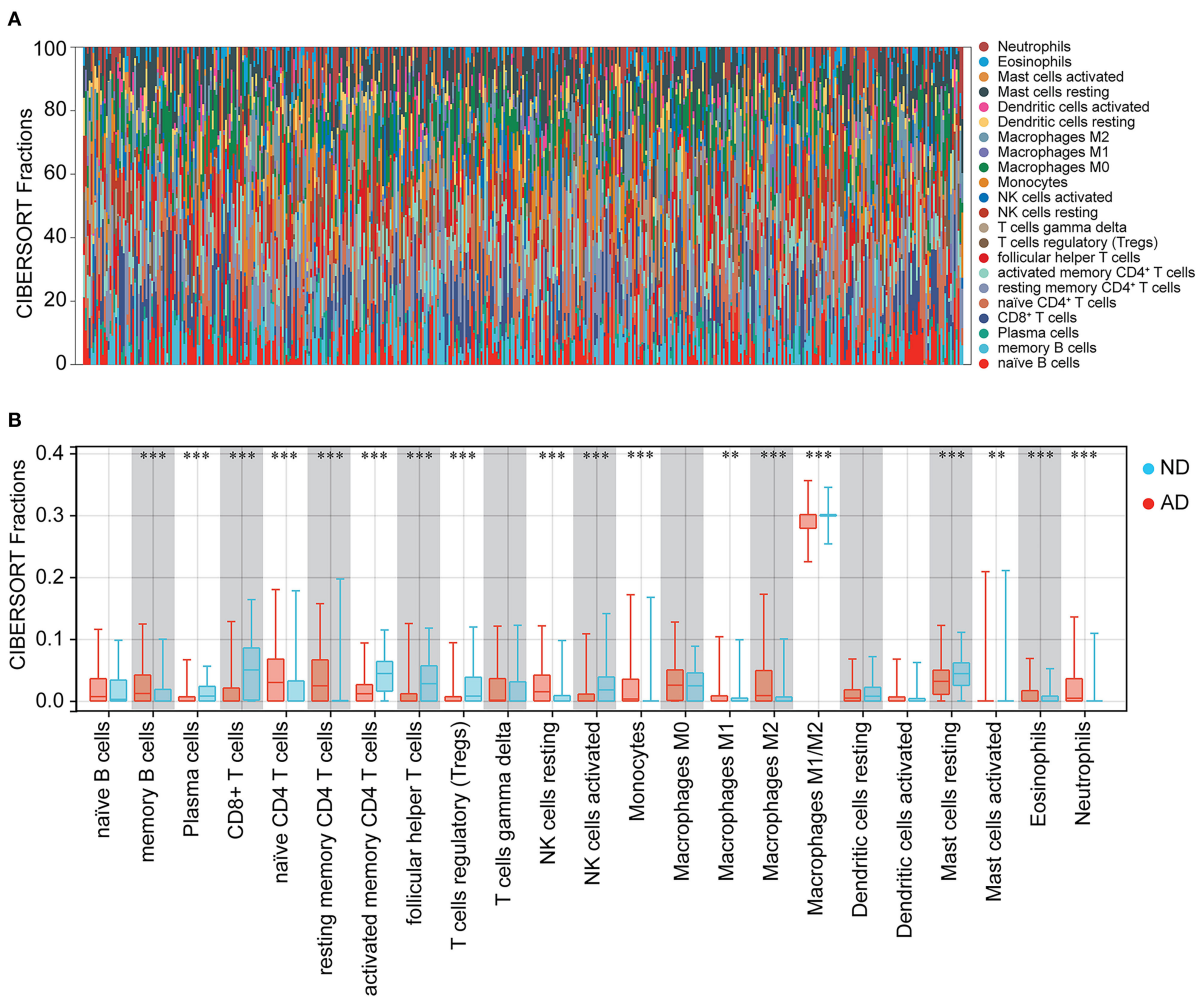
Immune cell infiltration between ND and AD samples. **(A)** The percentage of 22 immune cells in each sample. **(B)** Different levels of immune infiltrating cells between ND and AD samples.

Immune cells, extracellular matrix, and other factors in the body are important for clinical therapeutic sensitivity and disease diagnosis. In this study, we used CIBERSORT to compare the proportion of 22 immune infiltrating cells in ND and AD samples (Figure 8A). The immune cell infiltration was compared between AD and ND samples in the boxplot (Figure 8B). The results showed that memory B cells, naïve CD4^+^ T cells, resting memory CD4^+^ T cells, NK cells resting, macrophages M2, activated mast cells, eosinophils, and neutrophils were significantly higher in patients with AD. In contrast, plasma cells, CD8^+^ T cells, activated memory CD4^+^ T cells, follicular helper T cells, regulatory T cells (Tregs), activated NK cells and resting mast cells were significantly lower in patients with AD.

**Figure 9:**
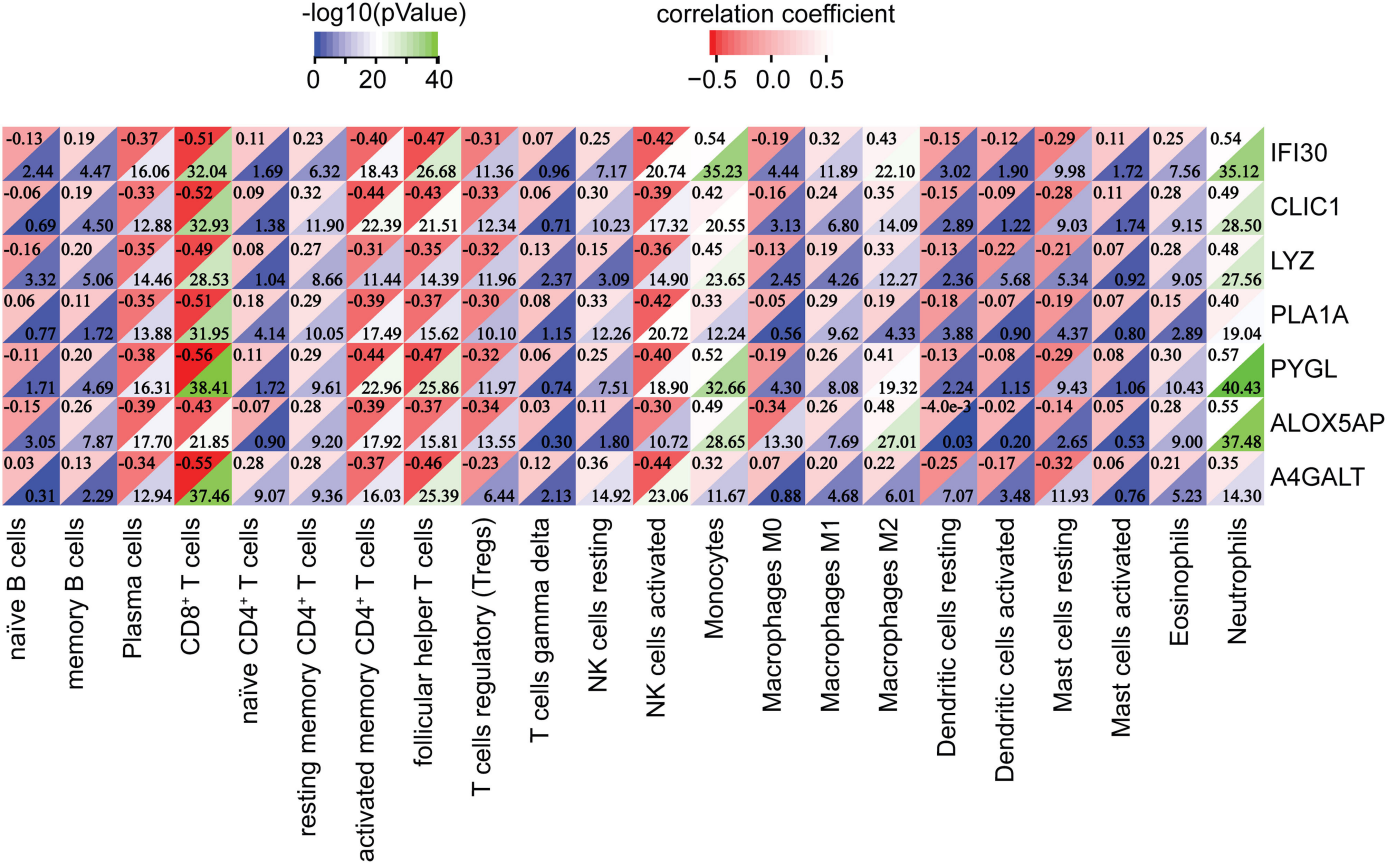
Correlation between the hub genes and immune infiltrating cells. **p < 0.01, ***p < 0.001.

Subsequently, the relationship between hub genes and immune infiltration was analyzed. The hub genes were significantly negatively associated with plasma cells, CD8^+^ T cells, activated memory CD4^+^ T cells, follicular helper T cells, Tregs, activated NK cells, and resting mast cells, while the opposite was true with resting memory CD4^+^ T cells, resting NK cells, monocytes, macrophages M1, macrophages M2, and neutrophils (Figure 9). The results suggest that the hub genes may play an important role in the immune microenvironment.

### Drug from DrugBank

In this study, we searched the DrugBank database for drugs targeting seven cuproptosis genes (Table 1). The FDA has approved six of these drugs; several others are being investigated intermittently; and one drug has been discontinued. There are no studies available for the *A4GALT, PLA1A*, and *PYGL* genes. It is believed that copper (DB09130) does have an effect on the *CLIC1* gene and is mostly used in emergency contraception, non-traceable elements, and dietary supplements, although the exact mechanism is unknown due to the wide spectrum of enzymes that use copper ions as co-factors. Acting as a ligand for *CLIC1* target genes, Artenimol (DB11638) also acts as a regulator of the cell cycle and inserts into membranes to form chloride channels at appropriate pH levels (34). In tissues and body fluids, sucrose (DB02772) acts as a nutritional supplement to Lysozyme C (encoded by the LYZ gene), which has an important role in enhancing immune responses (35, 36). As a feed additive, arsanilic acid (DB03006) is a toxic compound containing arsenic that induces blindness in animals. It has received approval for use in veterinary medicine for treating intestinal diseases in pigs and poultry (36). It is known that propyl alcohol (DB03175) is one of the best targets for lysozyme C and is primarily used for skin disinfection as well as a preservative that can be used in both clinical and domestic settings (35). Known as part of the non-essential amino acid family, aspartic acid (DB00128) can act on lysozyme C, but it needs to be studied further. Rose bengal (DB11182) is used as a diagnostic agent and is recommended for eye examinations, cornea, or conjunctiva (37) (Supplementary Table S2).

**Table 1:** Drugs targeting these seven hub genes obtained from the DrugBank database.

**Gene symbol**	**Protein**	**UniProt ID**	**DrugBank ID**	**Name**	**Drug group**	**Actions**
IFI30	Tryptophan-tRNA ligase, cytoplasmic	P23381	DB00150	Tryptophan	Approved, nutraceutical, withdrawn	Inhibitor
			DB01831	Tryptophanyl-5'amp	Experimental	
			DB04537	Tryptophanamide	Experimental	
A4GALT						
ALOX5AP	Arachidonate 5-lipoxygenase-activating protein	P20292	DB05225	AM103	Investigational	Inhibitor
			DB04929	DG031	Investigational	
			DB06346	Fiboflapon	Investigational	
			DB16739	MK-886	Experimental	Inhibitor
			DB16346	Veliflapon	Investigational	Inhibitor
CLIC1	Chloride intracellular channel protein 1	O00299	DB09130	Copper	Approved, investigational	
			DB11638	Artenimol	Approved, experimental, investigational	Ligand
LYZ	Lysozyme C	P61626	DB02772	Sucrose	Approved, experimental, investigational	
			DB03006	Arsanilic acid	Experimental, vet_approved	
			DB03120	p-Toluenesulfonic acid	Experimental	
			DB03189	Cu-Cyclam	Experimental	
			DB03487	(S)-Aspartimide	Experimental	
			DB02759	4-methyl-umbelliferyl-N-acetyl-chitobiose	Experimental	
			DB03013	N-acetyl-beta-D-glucosaminyl-(1->4)-N-acetyl-beta-D-glucosamine	Experimental	
			DB03175	Propyl alcohol	Approved	
			DB02159	(R)-Propylene glycol	Experimental	
			DB04194	Triacetylchitotriose	Experimental	
			DB04268	Methylumbelliferyl chitotriose	Experimental	
			DB00128	Aspartic acid	Approved, nutraceutical	
			DB06912	UNDECA-3,7-DIENE-1,3,7,11-TETRACARBALDEHYDE	Experimental	
			DB03967	Dodecyl sulfate	Experimental	
			DB11182	Rose bengal	Approved, investigational	ligand
PLA1A	Phospholipase A1 member A	Q53H76				
PYGL	Glycogen phosphorylase, liver form	P06737				

## Discussion

In this study, seven cuproptosis genes were screened for association with AD using the public dataset GSE33000. GO analysis showed that they were involved in the phosphoribosyl pyrophosphate and lipoxin biological processes, as well as the cellular lipid metabolic process and glycogen catabolic process. There is no doubt that elevated levels of PRPP in humans are associated with an excess of uric acid production and accumulation, which is negatively correlated with Alzheimer's disease prevalence (38). Neuroinflammation may be beneficial for patients with early-onset Alzheimer's disease, and it may help reverse or at least slow tau protein accumulation in the brain, preventing dementia (25). By helping macrophage differentiation and activation, lipoxin, derived from arachidonic acid, could reduce the immune response in early patients with AD by releasing cytokines that decrease inflammation (39, 40). Based on these cuproptosis genes, prediction models were developed and validated using the GSE48350, GSE5281, and GSE15222 datasets, respectively. Furthermore, GSEA demonstrated that all of these pivotal genes were associated with neurodegenerative diseases, particularly AD, which validated these gene selections to some extent (Figure 6).

The diagnostic models developed from four of the seven cuproptosis genes (including IFI30, PLA1A, ALOX5AP, and A4GALT) can serve as a guide to the clinical diagnosis of AD. An antigen-presenting cell (APC)-expressing gamma-interferon-induced lysosomal thiol reductase (GILT, encoded by IFI30) reduces disulfide bonds through endocytic proteins, presenting immunogenic peptides bound to the major histocompatibility complex (MHC) class II (41). Consistent with our findings that the IFI30 gene is highly expressed in patients with AD, GILT is highly expressed in microglia surrounding Aβ and is involved in Aβ clearance (42, 43). Abnormalities of lipid metabolism in the brain are characteristic of AD (44, 45). It is possible to reduce the risk of AD by converting phosphatidylcholine to lysophosphatidylcholine-DHA in liver tissues and transporting it into the brain across the blood-brain barrier (46, 47). The 5-lipoxygenase-activating protein (FLAP, encoded by ALOX5AP) is widely distributed in the central nervous system and functions to regulate the activation of the 5-lipoxygenase enzyme (48). It has been shown that selective pharmacological inhibition of FLAP significantly reduces Aβ levels and deposition in amyloid precursor protein (APP) transgenic mice (49) and regulates endogenous tau metabolism *in vivo* (50). A4GALT is involved in regulating the synthesis of glycosphingolipids, which is associated with several neurodegenerative diseases, including AD and Parkinson's disease (36, 37). Neuroinflammation is more prevalent in patients with mild cognitive impairment and AD. There is research showing that a combination therapy that reduces amyloid plaque formation and limits neuroinflammation may be more effective than treating either alone (51).

Here, we further explored the level of infiltration of immune infiltrating cells in patients with AD. The results showed that the M0 macrophages were not significantly different in both AD and ND, while the M1/M2 macrophages were significantly lower in AD. According to the results, increased activation and differentiation of M0 macrophages into M2 macrophages reduces inflammation and contributes to delaying the onset of AD. Tregs, the subset of CD4^+^ T cells, are essential for maintaining immune homeostasis and downregulating patients with AD. The results have shown that early depletion of Tregs is associated with accelerated cognitive impairment and that restoring Tregs reduces Aβ deposition and improves cognition, but the results of this study remain controversial (52). Neutrophils are also upregulated in AD. A significant increase in neutrophils was found in AD brains and AD model mice, and hyperactivity of neutrophils is associated with AD (53). It was found that neutrophils may play a role in AD as they produce large quantities of reactive oxygen species, thereby causing AD and cognitive decline through the LFA-1 integrin (25).

The gamma interferon-inducible lysosomal thiol reductase (GILT, encoded by IFI30) is expressed in antigen-presenting cells (APCs), such as dendritic cells, monocytes/macrophages, and B cells, and it is highly expressed in microglia in the AD brain (42). The chloride intracellular channel 1 (CLIC1) protein, a potential marker of neurodegenerative processes, is significantly increased in peripheral blood mononuclear cells of patients with AD (54). The LYZ gene, a significant member of non-specific immunity, is upregulated in the cerebrospinal fluid (CSF) of patients with AD and inhibits the appearance of toxic Aβ oligomers (55). Abnormal brain glucose catabolic processes are associated with the formation of amyloid plaques in the brain and the onset of memory loss (56). In contrast, the relationship between PYGL (glucose metabolizing enzyme) and AD remains unclear.

Finally, the drugs targeting the above genes were retrieved from the DrugBank database. An excess of copper (DB09130) acts as a catalyst for a variety of biological processes (57), accumulating in neurofibrillary tangles and regulating APP gene expression (45, 46). Aβ peptide interaction with copper and other metals is thought to promote gain-of-function activity and lead to neurotoxicity (15). As an immunomodulator and neuroinflammatory drug, Artenimol (DB11638) inhibits neuronal apoptosis, modulates Tau autophagy, and protects AD mice from neuronal damage (58–60). There is no doubt that sucrose (DB02772) is a valuable nutritional supplement, but excessive consumption can lead to type 2 diabetes, and type 2 diabetes is associated with a high incidence of Alzheimer's disease (61). There is an increase in LYZ in the brains of transgenic mice and humans with AD, pointing to new therapeutic strategies to slow its progression (55). Propyl alcohol (DB03175) acts on the LYZ target, but its function still needs further study. In addition to its functionality in protein synthesis, aspartic acid (DB00128) is also involved in the urea cycle and gluconeogenesis and affects AD indirectly (62, 63). There is a xanthene dye, rose bengal (DB11182), which has been used to treat colon cancer (64). Furthermore, rose bengal inhibits the toxic effects caused by Aβ aggregation (65) and has a therapeutic effect on Tau aggregation (66).

## Conclusion

In summary, we investigated seven cuproptosis genes and AD-related hub genes by using bioinformatics techniques. The biological role of these genes in AD development was explored. Further experiments are needed to confirm the function. Based on logistic regression analysis, we constructed a diagnostic model that can diagnose patients with AD by detecting the expression of several genes in the brain tissues. Additionally, immune cells expressed themselves more strongly in AD, indicating that they may be crucial to the immunological microenvironment. However, further research is needed to explore their specific effects. Currently, only a few drugs targeting these pivotal genes are predicted to alleviate AD, suggesting that additional drugs need to be developed.

## Data availability statement

The original contributions presented in the study are included in the article/Supplementary material, further inquiries can be directed to the corresponding authors.

## Author contributions

EZ and FD contributed to the conception, design of the study, and wrote the first draft of the manuscript. TC and SL organized the database. EZ, FD, and CX performed the statistical analysis. XS wrote sections of the manuscript. All authors contributed to the manuscript revision and read and approved the submitted version.

## Supplementary material

Supplementary Figure 1 Expression of hub genes in the AD and ND samples GSE15222, GSE48350, and GSE5281.

Supplementary Figure 2 Protein-protein interaction of seven hub genes.

Supplementary Table 1 Differentially expressed genes between AD and ND samples in GSE33000.

Supplementary Table 2 Information on drugs targeting these seven hub genes.
