# Supplementary figures and images for: Diagnostic models and predictive drugs associated with cuproptosis hub genes in Alzheimer's disease
Source: Front Neurol. 2023 Jan 26;13:1064639. doi: 10.3389/fneur.2022.1064639 (PMC9909238; doi:10.3389/fneur.2022.1064639)

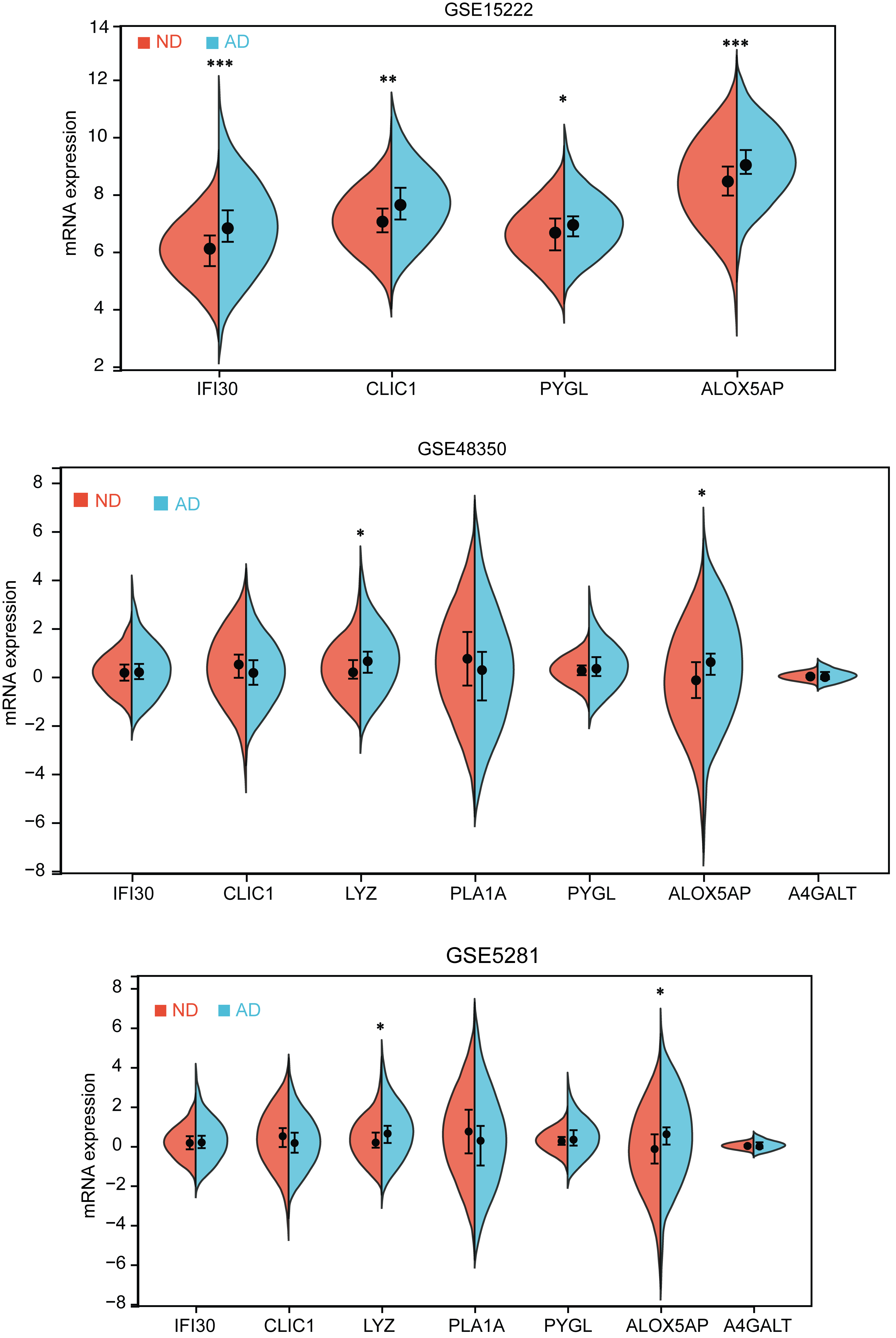

Supplement: Supplementary Figure 1 — Expression of hub genes in the AD and ND samples GSE15222, GSE48350, and GSE5281. [file Image_1.TIF]

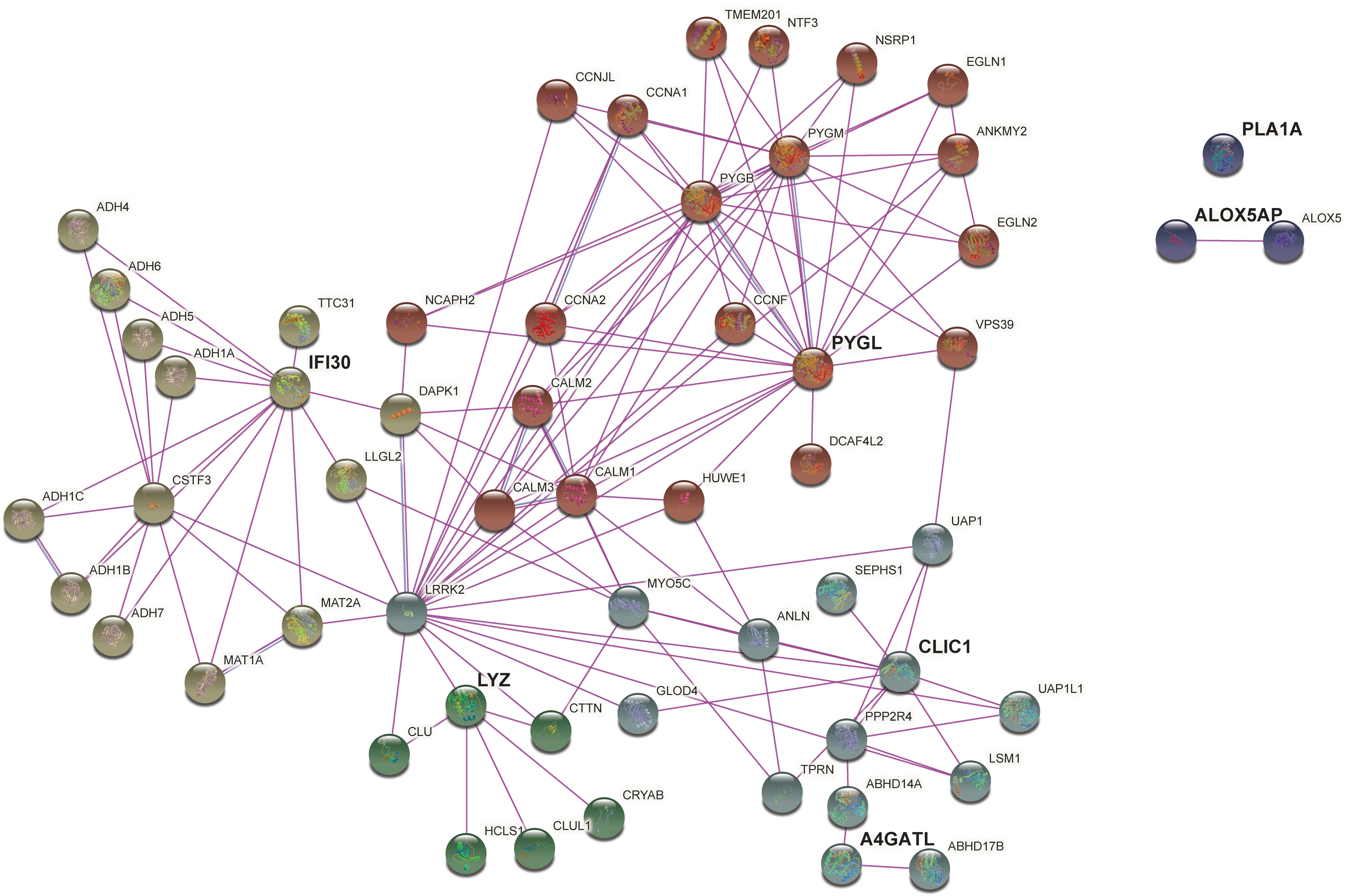

Supplement: Supplementary Figure 2 — Protein-protein interaction of seven hub genes. [file Image_2.TIF]
