# Supplementary material for: Diagnostic models and predictive drugs associated with cuproptosis hub genes in Alzheimer's disease
Source: Front Neurol. 2023 Jan 26;13:1064639. doi: 10.3389/fneur.2022.1064639 (PMC9909238; doi:10.3389/fneur.2022.1064639)
Supplement: Supplementary Table 2 — Information on drugs targeting these seven hub genes. [file Table_2.docx]

| **Table S2**  **Information on drugs targeting these hub genes.** | | |
| --- | --- | --- |
| **DrugBank ID** | **Name** | **Indication/Associated Conditions** |
| DB00150 | Tryptophan | Acute Renal Failure (ARF) |
|  |  | Depression |
|  |  | Renal Failure, Chronic Renal Failure |
| DB05225 | **AM103** | Asthma |
|  |  | Cardiovascular |
| DB04929 | **DG031** | Heart disease |
|  |  | Myocardial infarction |
| DB06346 | **Fiboflapon** | Inflammatory disorders (unspecified) |
| DB09130 | Copper | Emergency Contraception |
|  |  | IUD therapy |
|  |  | Trace Element Deficiency |
|  |  | Dietary supplementation |
| DB11638 | **Artenimol** | Malaria caused by Plasmodium falciparum |
| DB02772 | Sucrose | Nutritional supplementation |
| DB03175 | **Propyl alcohol** | Skin disinfection |
